# DNA in a bottle—Rapid metabarcoding survey for early alerts of invasive species in ports

**DOI:** 10.1371/journal.pone.0183347

**Published:** 2017-09-05

**Authors:** Yaisel J. Borrell, Laura Miralles, Hoang Do Huu, Khaled Mohammed-Geba, Eva Garcia-Vazquez

**Affiliations:** 1 Department of Functional Biology, University of Oviedo, Oviedo, Spain; 2 Department of Aquaculture Biotechnology, Institute of Oceanography, Vietnam Academy of Science and Technology, Nha Trang, Vietnam; 3 Genetic Engineering and Molecular Biology Division, Faculty of Science, Menoufia University, Egypt; University of Hyogo, JAPAN

## Abstract

Biota monitoring in ports is increasingly needed for biosecurity reasons and safeguarding marine biodiversity from biological invasion. Present and future international biosecurity directives can be accomplished only if the biota acquired by maritime traffic in ports is controlled. Methodologies for biota inventory are diverse and now rely principally on extensive and labor-intensive sampling along with taxonomic identification by experts. In this study, we employed an extremely simplified environmental DNA (eDNA) sampling methodology from only three 1-L bottles of water per port, followed by metabarcoding (high-throughput sequencing and DNA-based species identification) using *18S* rDNA and *Cytochrome oxidase I* as genetic barcodes. Eight Bay of Biscay ports with available inventory of fouling invertebrates were employed as a case study. Despite minimal sampling efforts, three invasive invertebrates were detected: the barnacle *Austrominius modestus*, the tubeworm *Ficopomatus enigmaticus* and the polychaete *Polydora triglanda*. The same species have been previously found from visual and DNA barcoding (genetic identification of individuals) surveys in the same ports. The current costs of visual surveys, conventional DNA barcoding and this simplified metabarcoding protocol were compared. The results encourage the use of metabarcoding for early biosecurity alerts.

## Introduction

Biosecurity issues derived from introduced biota are increasing concerns in the marine realm because precious marine biodiversity is at risk [1, 2, 3, 4]. In addition, the introduction of non-indigenous species has economic consequences because it may affect seafood production. For example, the invasive species *Crepidula fornicata* almost destroyed the oyster farms in Brittany [5]. Ports and marinas are perhaps the keystones in maritime biosecurity [6, 7, 8, 9, 10]. They are hubs of maritime traffic where vessels of all the continents stop for days or months. Therefore, the accompanying biota may leave the vessel, settle down in the port and eventually depart for other areas on other ships. Ballast water [11], hull fouling [12, 13] and even bilge water [14] are the main ship compartments where accompanying biota can survive. In ports, ships may clean hulls, ballast tanks and decks, which inadvertently liberates undesired species.

Early stage detection of introduced non-indigenous species (NIS) in ports has been claimed to be a priority and can be done through biota surveys. However, modern biota surveys rely mainly on classic sampling and visual taxonomic identification of biota. There are different sampling protocols recommended for port surveys [15, 16, 17, 18], and all of them are based on final taxonomic assessment from experts. Recent innovations in this field include DNA analysis. The individuals who are sampled may exhibit ambiguous phenotypes, especially in species with phenotypic plasticity and cryptic species that may make recognition through classic taxonomic methodology difficult. DNA can remove the ambiguity of their taxonomic status in these cases. DNA barcoding, i.e., the use of a consensus gene for individual genetic species identification, is increasingly being employed in marine settings and port biota surveys [9, 19, 20, 21].

One step beyond classic DNA barcoding is metabarcoding [10, 19, 22, 23, 24]. In its simpler version, it consists of extracting DNA from mass collections or environmental samples (generally water or sediments), then amplifying and sequencing one barcode gene using Next Generation Sequencing (NGS). This method allows to researchers to obtain thousands of sequences at the same time. After a relatively complex bioinformatics analysis, the sequences can be assigned to operational taxonomic units (OTUs), and the OTUs are compared to a reference database to determine the specimen’s taxonomic classification [25, 26].

Some drawbacks of metabarcoding include that still has relatively high costs and the bioinformatics involved are complex [27]. If the method relies on PCR amplification, possible primer-biased preferential amplification for some taxa may obscure the results by artificially enriching a limited set of taxonomic groups [28]. On the other hand, some barcodes may not be adequate for solving taxonomic identities in several groups of organisms because they are too conserved in such groups and cannot distinguish between related species [25, 29]. Moreover, metabarcoding is not a quantitative method yet although some progress on this goal has been made using different approaches (e.g., [30]). These problems can be solved using different primers and targeting different genes, increases the cost of metabarcoding though. Finally, since marine water masses are enormous and highly dynamic due to currents and tidal movements, the eDNA of scarce species may be highly diluted and high volumes of water samples are employed for metabarcoding, as large as 100 L [10, 27]. This may be a practical problem for routine surveys because such a large volume of water cannot be easily refrigerated or frozen until analysis, so should be filtered in situ or frozen [31, 32, 33].

For some problematic species that are either invasive, elusive or endangered, species-specific markers have been designed that can be used directly from eDNA (e.g., probe-based qPCR assays) and are highly sensitive (e.g., [34, 35, 36]). However, for exploratory purposes and full biota inventories, this method is not adequate because each marker targets a single species.

In this study, we have approached the potential utility of metabarcoding as an exploratory method for an early alert of invasive species based on an extremely simplified and easy sampling protocol of 3 L of water. If successful, it could be used in routine surveys by managers and port staff. Minimal analysis was done in the laboratory and the rest was externalized, including PCR amplification of two genes and bioinformatics. Costs/benefits analysis are essential when different methodologies, including novel ones, are proposed to solve a biological problem [27]. Therefore, metabarcoding costs were compared to the costs of a classic sampling and DNA barcoding survey of fouling invertebrates conducted in the same locations [9].

## Materials and methods

### Sampling and sampling locations

**Fig 1:**
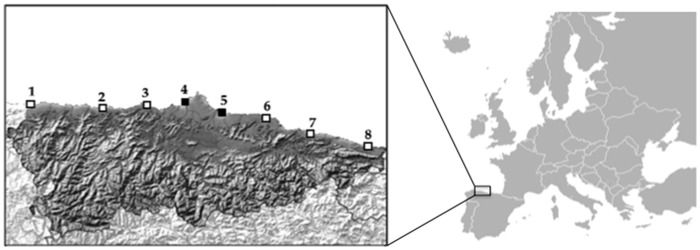
Map showing the eight ports analysed in this study. International cargo ports are marked with black squares and fishing ports and marinas with white squares. Numbers from 1 to 8 are Figueras (Eo), Luarca, Cudillero, Aviles, Gijon, Villaviciosa, Ribadesella and Luarca.

In a previous study, a DNA barcoding survey for intertidal fouling invertebrates was conducted in eight ports of different sizes and uses [9]. The considered ports were selected from West to East (Figueras, Luarca, Cudillero, Aviles, Gijon, Villaviciosa, Ribadesella and Llanes (Fig 1)) and are in the Asturias region (43°20′N 6°00′W) of the Cantabrian Sea coast (Bay of Biscay) in the northern Iberian Peninsula. Aviles and Gijon are commercial ports under national Spanish authority that receive large international cargo vessels and have adjacent fishing ports and marinas. The other six locations are fishing ports and the associated marinas are under Asturias regional authority, which oversees local maritime traffic, arrival of fishing catches (from national and international waters) and recreational boating [9].

In that study, artificial structures in three points within each port were sampled in a two-week sampling period. The detailed description of the sampling methodology can be found in [9]. For eDNA analysis, water samples were taken close to the surfaces previously sampled for classic taxonomy and DNA barcoding studies. All water samples were collected from public use portions of these ports. Therefore, no specific permissions were required for collection. Only the upper layer of the water mass (30 cm deep in water as in other studies, e.g., [31]) was sampled to avoid diving or complicating the sampling procedure. Sterile plastic bottles and gloves for preventing contamination with researcher’s DNA were used. In total, 24 1L water samples were taken from 3 sampling points in 8 ports, and the points were separated by approximately 200 m with one point near the port mouth, one in the inner section, and one-half way between those two points.

Water samples were cooled while they were transported to the lab and since DNA extractions could not be done in the field, the samples were immediately frozen at -20°C until DNA extractions was conducted as recommended [33]. The eDNA extractions were carried out 15 days later due to logistic and organization issues. Hinlo *et al*. [33] found no significant differences in eDNA yield recovery after freezing the water samples for four days and posterior DNA extractions using the PowerWater^®^ DNA Isolation Kit (MOBIO Laboratories, USA) with the DNeasy-Freeze combination, which recovered the highest eDNA yield out of five different methods tested to preserve and extract eDNA. At the same time, after 10 days of refrigerated or frozen storage of the samples, there were no significant differences among them in terms of eDNA yield although both methods showed decreases in the eDNA yield that was recovered [33].

### The eDNA extraction

After unfreezing the water samples at room temperature, they were filtered through a 0.2-μm Nuclepore^™^ membrane and DNA was extracted from the filters (1 filter by 3 L water sample). DNA extraction replicates could improve diversity estimates as well as the ability to separate samples with different characteristics [37]. In our study, we replicated samples (3) within the ports to increase the chances of detecting NIS. Total genomic DNA was extracted using the PowerWater^®^ DNA Isolation Kit (MOBIO Laboratories, USA), which yields high quality DNA for DNA barcoding or meta-barcoding applications. The manufacturer's instructions were followed. DNA extractions were made in an exclusive sterile room in a different building. Moreover, DNA extractions were conducted using negative controls and on different days for samples using sterile technique inside a laminar air flow chamber continuously disinfected by UV light, absolute ethanol and 10% bleach solution cleaning to prevent contamination. The DNA samples from ports were quantified using the Picogreen method and Victor-3 fluorometry. DNA samples were finally analyzed using two different metabarcodes.

### Polymerase chain reaction (PCR), massive sequencing and bioinformatics analyses

The PCR reactions were performed by Macrogen Korea using negative controls to monitor possible contamination as well as Roche FastStart^™^ High Fidelity Taq DNA Polymerase and the protocols described in the Amplicon Library Preparation Manual (Roche 2010; GS FLX Titanium Series). Geller *et al*. [38] primer pairs for the *Cytochrome Oxidase I* (COI) gene and Machida and Knowlton's [39] for the *18S* rRNA gene (18S, designated primers #1 and #2_RC) were used. Thermocycling conditions were 1x: 94°C for 3 min; 35x: 94°C for 15 sec, 55°C for 45 sec and 72°C for 1 min; and finally, 1x: 72°C for 8 min and 4°C on hold. Library construction included quality controls for size (Agilent Technologies 2100 Bioanalyzer using a DNA 1000 chip) and quantity (Roche's Rapid library-standard quantification solution and calculator). The bands of expected sizes (800 bp in COI and 500 bp in 18S) were sequenced in the 1/8 plate GS-FLX run (Roche/454 Life Sciences, Branford, USA).

The multiplexed reads were assigned to samples while accounting for their nucleotide barcodes (demultiplexing). Zero base errors were allowed in this sorting by a tag step. CD-HIT-OTU [40] was used to filter out erroneous and chimeric reads by combining sequence clustering and statistical simulations. Quality filters based on the characteristics of each sequence were applied to remove short (<100 bp) and low-quality reads (<20 Phred values) as well as extra-long tails. Primer pairs were trimmed. Filtered reads were aligned and clustered at 100% identity using CD-HIT-DUP, and the chimeric reads were identified and eliminated from the duplicate clusters (CD-HIT-OTU User's Guide (http://weizhong-lab.ucsd.edu/cd-hit-otu)). Secondary clusters were then recruited into the primary clusters, and the remaining representative reads from the non-chimeric clusters were grouped into OTUs using a greedy algorithm with a 97% cut-off for 18S sequences (e.g., at a species level following the method of Stackebrandt and Goebel [41] for ribosomal sequences) and 98% for COI sequences (see Ratnasingham and Hebert [42, 43] for useful discussions about this). This result was used to avoid false OTUs because of PCR errors, sequencing errors and other technical errors. The OTUs were then BLASTed against the NCBI database for the case of the COI reads with e-value threshold of 0.01, ≥97% sequence homology and >90% sequence coverage for accepting hits. The remaining 18S reads were aligned using UCLUST [44] in QIIME [45] and the SILVA database [46] was used for obtaining the OTU list.

The sequences of OTUs taxonomically assigned to genera of interest due to the occurrence of invasive species within a genus, and/or occurrence of species of a genus within the conventional sampling biota study [9], were extracted from the raw FASTA files and checked again against GenBank using conventional BLAST software. The identity, coverage and E-value with the best match reference sequence were retrieved. We followed WORMS [47] for taxonomic names and classification. The references and retrieved OTUs that used alternative nomenclature were named after WORMS in this study.

### Statistical analysis

The number of sequences (reads) of a multicellular species obtained from metabarcoding procedures is not proportional to the number of individuals of such species [30]. For this reason, metabarcoding data were scored as presence / absence for each OTU and measured as 1 / 0, respectively. PAST version 3 software [48] was used for obtaining Alpha-diversity measures used here: taxa-S (species richness or number of species, in this case OTUs), Margalef's richness index, and finally, Shannon-Weaver (H) (see Harper [49] for indices details). Global beta-diversities were also estimated through Whittaker and Routledge indices (details in Koleff *et al*. [50]) using the same software. Several studies have found that metabarcoding accurately recovers alpha-diversity (species richness) and beta-diversity (species turnover) information, in addition to generating the same management recommendations as morphological biodiversity datasets (e.g., [37, 51, 52]).

For a comparison of OTU results (presence/absence from the total list of OTUs obtained for the two genes) among the genes and the ports, a Non-metric Multidimensional Scaling analysis (MDS) and ANOSIM analyses were conducted, using Euclidean distances and 9 999 bootstraps using PAST version 3 [48]. At the same time, Scatter and Shepard plots were constructed in PAST to visualize the relationships among port OTUs found from the two DNA barcodes. Stress and squared r for the two axes were also calculated.

Comparisons between diversity indices of fouling invertebrates in the region (considering all ports together) obtained from the conventional sampling + DNA barcoding method used in the Miralles *et al*. [9] study and this work (simplified sampling (water) + metabarcoding) was done using only presence-absence data, and the metabarcoding subset of fouling organisms for comparable results. Alpha diversity indices were compared using Diversity Permutation Tests. This module computes several diversity indices for two samples and then compares the diversities using random permutations. A total of 9999 random matrices with two columns (samples) are generated, each with the same row and column totals as in the original data matrix (see manual of the PAST software). The analyses mentioned above were completed using the free software PAST version 3 [48].

### Cost estimates

Costs of barcoding and visual analysis of animal specimens have been previously estimated according to Spanish standards (i.e., [9, 53]), and the present calculations are based on them. The costs of labor (proportional part of the salary for the time dedicated to different tasks) were estimated from Spanish official technician wages for the salaries (Resolution 2000 *Boletín Oficial del Estado* 49 of 26 of February of 2015) since the study was carried out in Spain. Sampling water from each port took no longer than 30 minutes (10 minutes per sampling point within the port), while sampling invertebrates from each port from conventional methodology required approximately 6 hours (2 hours per sampling point).

For consumables and external services, the real costs of barcodes in Miralles *et al*. [9] were 5€/individual sample (extraction kit + PCR products + external sequencing services). For the metabarcodes obtained in this work the cost was 194€/sample (extraction kit + external services of library preparation, sequencing and bioinformatics). The travels for sampling between the ports and the laboratory were logically the same whatever analytical methodology was employed, and the results were therefore excluded from the comparative estimations.

## Results

The quantity of total DNA obtained from the 3-L water bottles obtained from each port ranged between 0.457 ng/μL and 5.552 ng/μL (Table 1). Amplicon Libraries after PCRs and posterior NGS analysis were conducted and data by samples are now accessible in Genebank BioProject IDs: SAMN07345428, SAMN07345429, SAMN07345430, SAMN07345431, SAMN07345432, SAMN07345433, SAMN07345434, SAMN07345435. NGS with COI primers provided a total of 164,563 reads and average length reads of 664.6 bps. The quality-check process removed sequences due to presence of short (34,997) and ambiguous sequences (6,726) as well as possible chimera and homopolymer appearances (2,732) and that filtering left 120,111 reads that passed the quality filters (mean length = 615.8 bp) from which a total of 24,945 OTUs were assigned down to a family, genus or species level (S1 Table). For 18S primers, the samples from the Cudillero and Villaviciosa ports failed to provide a reliable 18S amplicons library (Table 1). NGS analyses provided a total of 144,294 reads with a mean length of 405.1 bps from the eDNA of six ports. The quality-check process eliminated the following types of sequences: too short (48,685); ambiguous (2,034); chimeras/homopolymers (2,850). The quality-check process left 90,725 reads (mean length = 374.5 bps). A total of 8,490 OTU counts were assigned down to a family, genus or species level (S1 Table). Rarefaction curves for the two metabarcodes and samples are provided in S1 Fig.

**Table 1:** Environmental DNA (eDNA) samples (final volume 100uL) obtained from 3L water samples in the ports from Asturias (Northern Spain, Bay of Biscay).

Sample	eDNA Conc. (ng/ul)	COI Amplicon Library fragm 800bp-Conc. (molecules/ul)	18S Amplicon Library fragm 500bp-Conc. (molecules/ul)
Eo	3.649	1.62 X 10^10^	4.66 X 10^9^
Luarca	2.072	1.08 X 10^10^	3.72 X 10^9^
Cudillero	0.457	2.50 X 10^10^	-
Aviles	2.628	4.55 X 10^9^	4.29 X 10^9^
Gijon	6.262	5.69 X 10^9^	2.50 X 10^10^
Villaviciosa	0.606	1.25 X 10^10^	-
Ribadesella	1.611	1.97 X 10^10^	1.14 X 10^9^
Llanes	5.552	2.51 X 10^10^	2.17 X 10^10^

The—symbol means that not PCRs were obtained from these samples.

**Fig 2:**
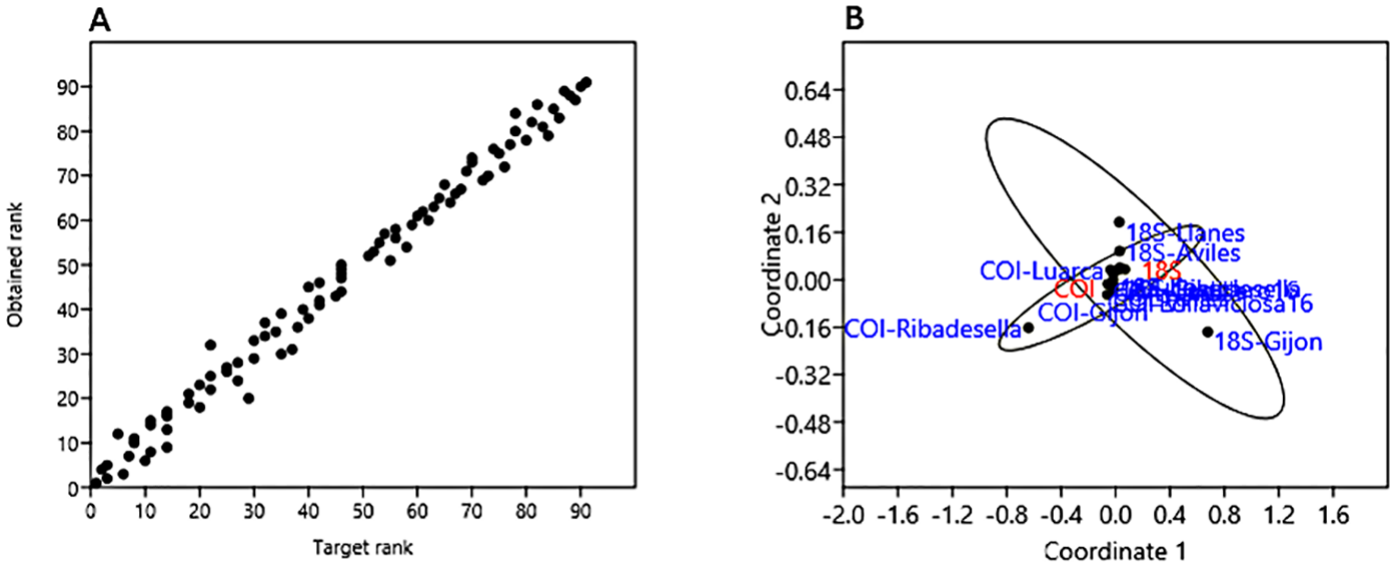
Non-metric Multidimensional Scaling of the metabarcodes found for *18S* rDNA and *Cytochrome oxidase I* gene in the analysed ports. A: Shepard plot; B: Scatter plot. The port names are given and genes acronyms are 18S for *18S* rDNA and COI for *Cytochrome oxidase I* gene. The 95% ellipsis for the data is shown in scatter plot.

The two DNA metabarcodes that were employed provided different taxonomic resolutions in this dataset (Table 2); COI yielded more taxa on average (mean OTUs per port of 26.30, SD 9.45) than 18S rDNA (mean 18.17, SD 15.95). The non-metric Multidimensional Scaling had a stress of 0.057 and r^2^ of axis 1 was 0.824 with the same value for axis 2 being 0.656 in the Shepard plot (Fig 2a). The COI metabarcodes of the analyzed ports were more similar to each other than 18S rDNA samples, and the samples grouped together in the MDS except for Ribadesella (Fig 2b) while the 18S rDNA results were more scattered, with some clear differences between Gijon and Llanes. In congruency with MDS analysis, alpha-diversities obtained in the eight ports for the two metabarcodes were quite different (ANOSIM p-value = 0.0006 after 9999 permutations) (Table 2). Gijon and Llanes had the most diverse Metabarcode for COI and 18S rDNA respectively, while the least diverse Metabarcode corresponded to Llanes and Ribadesella respectively. Whittaker and Routledge’s beta-diversities were 3.8081 and 0.4702, respectively.

**Table 2:** Alpha-diversities obtained for *18S* rDNA and *COI* gene metabarcodes in the ports studied in this work.

	Taxa S		Shannon		Margalef	
	COI	18S	COI	18S	COI	18S
Eo	35	7	3.555	1.946	9.563	3.083
Luarca	19	6	2.944	1.792	6.113	2.791
Cudillero	24	-	3.178	-	7.237	-
Aviles	18	20	2.890	2.996	5.882	6.342
Gijon	38	20	3.638	1.634	10.17	2.337
Villaviciosa	22	-	3.091	-	6.794	-
Ribadesella	39	8	3.157	1.386	8.189	1.924
Llanes	16	48	2.773	3.871	5.41	12.14

The species list obtained from each of the two metabarcodes as merged into a global taxa list. The differences between ports for the number of OTUs were marked because in Cudillero and Villaviciosa, only COI data were available. Taking into account only marine taxa, they ranged between 19 in Villaviciosa to 61 in Llanes (Table 3). Most taxa were plankton microalgae, such as diatoms and protozoans such as ciliates (Table 3). A few OTUs corresponded to vertebrates (fishes *Albula*, *Clinostomus*, *Cyprinella*, *Dypturus*, *Oregonychthys*; the Anatidae *Chloephaga*). Invertebrates, which are the main focus of this study about invasive species, were a minority in all the ports ranging between 4 in Luarca and Aviles to 12 in Eo (Table 3).

**Table 3:** Genera inferred from *18S* rDNA and *COI* metabarcodes in the eight ports analysed in this study. E: Eo; L: Luarca; C: Cudillero; A: Aviles; G: Gijon; V, Villaviciosa; R, Ribadesella; Ll: Llanes; Total, number of ports where the genus was inferred. In bold, genus containing exotics species. 0 = absence, 1 = presence.

	Genus	E	L	C	A	G	V	R	Ll	Total
Annelida	*Abarenicola*	0	0	0	0	0	0	0	1	1
	*Acanthostaurus*	0	0	0	0	0	0	0	1	1
	*Agaricomycetes*	0	0	0	1	0	0	0	1	2
	*Alaria*	0	0	0	0	1	0	0	0	1
Chordata, Actinopterygii, Albulidae	*Albula*	1	0	0	1	1	1	0	0	4
	*Amoebophrya*	0	0	0	0	0	0	0	1	1
Chordata, Squamata, Amphisbaenidae	*Amphisbaena*	1	1	0	0	0	0	0	0	2
	*Ancyromonas*	0	0	0	0	0	0	0	1	1
Nematoda, Chromadorea	*Aphelenchoides*	0	0	0	0	0	0	0	1	1
	*Aplanochytrium*	0	0	0	1	0	0	0	1	2
	*Archigregarinorida*	0	1	0	0	1	0	0	1	3
Entoprocta, Barentsiidae	*Barentsia*	0	0	0	1	0	0	0	0	1
	*Bathycoccus*	1	1	1	0	1	1	1	1	7
	*Bifurcaria*	0	0	1	0	0	0	0	0	1
	*Blastodinium*	0	0	0	0	0	0	0	1	1
Annelida	*Boccardiella*	0	0	0	0	0	0	0	1	1
	*Boeremia*	0	0	0	1	0	0	0	1	2
Annelida	*Capitella*	1	0	0	0	0	0	0	1	2
Gastrotricha, Chaetonotidae	*Chaetonotus*	0	0	0	0	0	0	0	1	1
Chordata, Aves, Anatidae	*Chloephaga*	0	0	0	0	0	0	0	1	1
	*Chlorella*	1	1	1	1	1	1	1	1	8
	*Chlorococcum*	0	0	0	1	0	0	0	0	1
	*Choreotrichia*	0	0	0	1	1	0	0	1	3
	*Chrysochromulina*	0	0	0	0	0	0	0	1	1
Chordata, Actinopterygii, Cyprinidae	*Clinostomus*	1	1	0	1	1	1	1	1	7
Chordata, Ascidiacea, Styelidae	*Cnemidocarpa*	0	0	0	0	1	0	1	0	2
	*Cymbella*	1	0	0	0	0	0	0	0	1
Chordata, Actinopterygii, Cyprinidae	*Cyprinella*	0	0	1	0	0	0	0	0	1
	*Desmodesmus*	0	0	0	1	0	0	0	0	1
	*Dicranum*	1	0	1	0	1	0	1	0	4
	*Dictyopteris*	0	0	0	0	1	0	0	0	1
	*Dicymbium*	1	1	0	1	1	0	1	1	6
Chordata, Chondrichthyes, Rajidae	*Dipturus*	0	0	0	0	1	0	0	0	1
	*Dolichomastix*	1	1	0	0	1	0	1	1	5
**Arthropoda, Cirripedia, Chthamalidae**	***Austrominius***	**0**	**0**	**0**	**1**	**0**	**0**	**0**	**0**	**1**
	*Emiliania*	0	0	0	1	0	0	0	1	2
	*Eugregarinorida*	0	0	0	0	1	0	0	0	1
	*Euplotia*	0	0	0	0	0	0	0	1	1
Arthropoda, Malacostraca, Lyssianasidae	*Eurythenes*	1	0	1	0	1	1	1	0	5
**Annelida, Polychaeta, Serpulidae**	***Ficopomatus***	**0**	**0**	**0**	**0**	**0**	**0**	**0**	**1**	**1**
	*Frustulia*	1	1	1	1	1	1	1	1	8
	*Geminigera*	0	0	0	0	0	0	0	1	1
	*Gonyostomum*	1	0	1	1	1	1	1	0	6
	*Grammonema*	1	1	0	1	1	0	1	0	5
	*Gregarinasina*	0	0	0	0	0	0	0	1	1
	*Gymnodinium*	0	0	0	0	0	0	1	1	2
	*Gyrodinium*	1	1	0	1	1	0	0	1	5
	*Haptoria*	0	0	0	1	0	0	0	1	2
	*Hazardia*	0	0	0	0	1	0	0	0	1
Echinodermata, Echinoidea, Echinometridae	*Heliocidaris*	1	0	0	0	0	1	1	0	3
	*Hypotrichia*	0	0	0	0	0	0	0	1	1
	*Kirchneriella*	1	0	1	0	1	1	0	0	4
Cnidaria, Anthozoa, Alcyoniidae	*Klyxum*	1	0	0	0	0	1	0	0	2
Mollusca, Gastropoda, Lacunidae	*Lacuna*	1	0	1	0	1	0	1	0	4
	*Landsburgia*	0	0	0	0	1	0	0	0	1
	*Mesodiniidae*	1	0	0	0	0	0	0	1	2
	*Micromonas*	1	1	1	1	1	1	1	1	8
	*Nannochloris*	0	0	0	0	0	0	0	1	1
	*Nannochloropsis*	1	1	1	1	1	1	1	1	8
	*Navicula*	0	0	0	0	0	0	0	1	1
	*Nebela*	0	1	0	0	1	0	1	0	3
	*Nitzschia*	0	0	0	0	1	0	0	0	1
	*Odontella*	0	0	0	0	0	0	0	1	1
	*Oligohymenophorea*	0	0	0	0	0	0	0	1	1
	*Oligotrichia*	0	0	0	1	0	0	0	1	2
Chordata, Actinopterygii, Cyprinidae	*Oregonichthys*	1	0	1	1	1	1	1	1	7
	*Ostreococcus*	1	1	0	1	1	0	1	1	6
	*Paraphysomonas*	0	0	0	0	0	0	0	1	1
	*Peronospora*	0	0	1	1	1	0	1	0	4
	*Phyllopharyngea*	0	1	0	0	0	0	0	1	2
	*Phytophthora*	1	0	1	1	1	1	1	0	6
	*Pinnularia*	1	1	0	0	0	0	1	1	4
Echinodermata, Asteroidea, Asteriidae	*Pisaster*	1	0	1	0	0	1	1	1	5
**Annelida, Polychaeta, Spionidae**	***Polydora***	**0**	**0**	**0**	**0**	**0**	**0**	**0**	**1**	**1**
	*Prostomatea*	0	0	0	1	0	0	0	1	2
	*Protaspidae*	0	0	0	0	0	0	0	1	1
	*Psammodictyon*	0	0	0	0	0	0	0	1	1
	*Pseudoperonospora*	1	0	1	0	1	0	1	0	4
	*Pyramimonas*	0	0	0	1	0	0	0	1	2
	*Pythium*	1	1	1	1	1	1	1	1	8
	*Rhodomonas*	0	0	0	0	0	0	0	1	1
	*Scytosiphon*	1	1	0	1	1	0	1	0	5
Mollusca, Cephalopoda, Sepiolidae	*Sepietta*	1	1	1	1	1	1	1	1	8
Annelida, Polychaeta, Serpulidae	*Serpula*	0	0	0	0	1	0	0	0	1
	*Skeletonema*	0	0	0	1	0	0	0	1	2
Arthropoda, Malacostraca, Solenoceridae	*Solenocera*	1	1	0	0	1	0	1	0	4
	*Sordariomycetes*	0	0	0	1	0	0	0	0	1
	*Stereocladon*	1	1	1	1	1	1	1	1	8
Mollusca, Gastropoda, Strombidae	*Strombus*	1	0	1	0	1	1	0	0	4
	*Teleaulax*	0	0	0	1	0	0	0	1	2
Gastrotricha, Thaumastodermatidae	*Tetranchyroderma*	1	1	0	1	1	0	1	1	6
	*Tetraselmis*	0	0	0	0	0	0	0	1	1
	*Thalassiosira*	1	0	0	1	1	0	1	1	5
	*Ulva*	0	0	0	0	0	0	0	1	1
	*Umbelopsis*	0	0	1	0	0	0	0	0	1
Annelida, Polychaeta, Serpulidae	*Vermiliopsis*	0	0	0	0	0	0	0	1	1
Arthropoda, Maxillopoda, Balanidae	*Wanella*	1	1	0	0	0	0	0	0	2

Compared to two different methods (conventional sampling + DNA barcoding and simplified sampling + metabarcoding), we found that in the 18S rDNA metabarcodes three genera with potential invasive species were shared with the dataset obtained from conventional sampling of fouling invertebrates in the same sampling locations [9], including the barnacle *Austrominius* (old genus name was *Elminius*), the tubeworm *Ficopomatus* and the annelid worm *Polydora*. They were found in Aviles (*Austrominius*) and Llanes (*Ficopomatus* and *Polydora*) ports (Table 3). The sequences of these OTUs were retrieved from the metabarcoding FASTA files and compared online with the GenBank database using BLAST nucleotides. The sequences retrieved with the best match corresponded to the invasive species recorded from conventional sampling from the same ports by Miralles *et al*. [9], including *Austrominius modestus*, *Ficopomatus enigmaticus* and *Polydora triglanda* (Table 4). These three species represented, on average, approximately 20.8% of the invertebrate species found in the two ports from metabarcoding (38 OTUs in total). The average percentage of individuals of these species in the same ports found through conventional barcoding in the Miralles *et al*. [9] study was similar (19.2%).

**Table 4:** Analysis of sequences identified as NIS invertebrates in the metabarcoding datasets. Showing results of BLAST analysis and sequence lengths, GenBank accession numbers of the best match reference, identity, query coverage and E-value.

	BLAST results of NGS sequences				
Species	Length	Best match	Identity	Coverage	E-value
*Austrominius modestus*	573	AY520635.1	100%	100%	8.00E-160
*Ficopomatus enigmaticus*	368	DQ317115.1	100%	100%	1.00E-98
*Polydora triglanda*	363	JN048723.1	99%	100%	1.00E-96

The diversity of fouling invertebrates found in this study for all the ports together was indeed higher when measured from specific sampling of fouling biota + DNA barcoding (S2 Table; data of fouling biota sampling were taken from Miralles *et al*. [9]) compared to the simplified protocol (water) + metabarcoding employed here (Table 5). The difference, however, was not statistically significant from the diversity permutation test p-values (Table 5).

**Table 5:** Alpha-diversities obtained at regional level (the eight ports together) for simplified sampling + metabarcoding (= metabarcoding; two metabarcodes combined) and for conventional sampling + barcoding analysis (= barcoding; calculated from Miralles *et al*. [9]). Permutation P values for the comparison of the regional diversity estimates using Diversity permutation test available in PAST version 3 (Perm p, 9 999 permutations).

	Metabarcoding	DNA Barcoding	Perm p
Taxa S	30	77	0.1505
Shannon H	3.401	4.344	0.2532
Margalef	8.526	17.5	0.1505

The costs of the two methods were estimated from Spanish official technician wages for the salaries (the study was carried out in Spain), in 8-h working days and the real costs from 2016 for barcodes and metabarcodes (Tables 6 and 7). The travels between the ports and the laboratory are the same and were excluded from the calculations. Sampling water from each port took no longer than 30 minutes (10 minutes per sampling point within the port), while sampling each port from conventional methodology needed approximately 6 hours (2 hour per sampling point). The total cost estimated for 671 barcodes was approximately 6,701 EU (~10 EU by sample) in the work by Miralles *et al*. [9]. Metabarcoding costs were split here into molecular analysis and bioinformatics, and they were 2,722.0 EU in total (for detecting 102 different taxonomical identities and 33,435 OTUS in 14 samples (8 using COI +6 samples using 18S rDNA as barcodes)). The total sum was higher for conventional sampling + barcoding.

**Table 6:** Time and labour costs estimates required for the identification of the 38 individuals of the three exotic species found in this study (n: number of individuals of each species) using: visual identification; conventional sampling and DNA barcoding; and simplified sampling (water) + metabarcoding.

Time estimates						
*Species*	*n*	Visual	DNA Barcoding			Metabarcoding
		*inspection*	*Tissue sampling*	*DNA Extraction*	*PCR preparation*	*DNA extraction from water*
*Austrominius modestus*	1	10 min x 1 = 10min	2 min x 1 = 2min	150 min	30min	45min
*Ficopomatus enigmaticus*	36	10 min x 36 = 360min	2 min x 36 = 72min			
*Polydora triglanda*	1	10 min x 1 = 10min	2 min x 1 = 2min			
***Sampling time in the port***		240 min		240min		30min
**Total time**		620 min		494 min		75 min
**Estimated cost**		413.5€		329.5€		50€

**Table 7:** Costs of consumables/external sequencing for three different methods used for the identification of the 38 individuals of the three exotic species found in this study (adapted from Ardura *et al*. [53]). Spanish salaries for laboratory technicians were taken from the official Resolution 2000 BOE 49 of 26 of February of 2015.

Cost of consumables and external analyses			
	Visual	DNA barcoding	Metabarcoding
	Fixative	Extraction kit	Extraction kit
		PCR products	Library/Sequencing/ Bioinformatics
		Sequencing	
**Estimated cost**	1€ x 38 = 38€	5€ x 38 = 190€	194€* x 2 (two ports)
**Total cost**	**451.5€**	**519.5€**	**438€**

* Cost by metabarcoding sample

A rough approximation was conducted to estimate the cost-benefit efficiency of each method for finding exotic species. We have estimated the cost for the identification of 38 exotics specimens of the three exotic species found in the present study using three different methods. The methods included Visual (morphology-based identification by a specialized taxonomist), conventional sampling + DNA barcoding (as in Miralles et al. [9]) and simplified sampling (water) and metabarcoding (this study) used as references for previous cost estimations (i.e., [9, 53]) (Tables 6 and 7). Visual identification required more time for sampling + analysis (620 min) than barcoding (494 min), and metabarcoding required less sampling effort and laboratory processing with a total of 75 min (Table 6). In contrast, consumables and sequencing analyses were more expensive for metabarcoding than for the two other methods (Table 7). Considering the time required for the analysis, consumables, and external sequencing (metabarcoding) the total cost estimates were 451.5 EU, 519.5 EU and 438 EU for visual, DNA barcoding and simplified sampling + metabarcoding (this work), respectively (Table 7).

## Discussion

This study provides evidence of the utility of a very simple metabarcoding-based methodology for detecting marine exotic species, even if they are at very low densities, as was the case for *Austrominius modestus* in Aviles and *Polydora triglanda* in Llanes where only one individual of each species was found in the visual sampling [9]. In this study, we found three NIS (38 OTUs) from 3-L water samples collected only once at each port without resampling. The same species were found in the visual and barcoding surveys in the same ports [9]. The cost of metabarcoding did not significantly surpass the other method (classic sampling and DNA barcoding) (Table 7), and the technical expertise required for the laboratory analysis carried out by the researchers was minimal. These facts support recommending the use of a metabarcoding approach for routine surveys in ports, but currently it would probably work best as an exploratory method for an early alert system. It is worth mentioning that the number of individuals cannot be fully determined using metabarcoding, which only counts DNA molecules. Despite this limitation, it seems this issue will not persist for long. Quantitative metabarcoding will likely become feasible in the near future [30, 54, 55]. Currently, the biodiversity based on individual counts cannot be properly estimated and only the presence of a species can be confirmed. For this reason, this technique is increasingly being employed for biodiversity inventories and should not be used as a way to compensate for the current decrease in the number of taxonomic experts [27, 53, 56]. The discipline of taxonomy is needed now more than ever now, especially for marine biota. DNA databases of references, such as GenBank and BOLD (Barcoding of Life Diversity) [42] rely on good complete taxonomic information for the voucher specimens. The absence of such information is a drawback of current barcoding projects and hampers the use of DNA-based methodologies (e.g., [21, 57, 58]).

Although metabarcoding has been recommended for port surveys [59, 60, 61] some improvements are necessary. One improvement should be the use of different types of environmental samples, not only water. In our study, the water samples that were analyzed primarily contained plankton species, which is logical because the water was sampled from the sea surface. Surveys of potential biological invasions should also consider fouling biota [10]. Sediments should be sampled from port walls as well (both artificial surfaces and natural rocks) for detecting early adherence of fouling individuals [62]. Moreover, it seems that eDNA is better preserved in sediments than in water [63]. Another improvement would be to conduct more extensive sampling, including targeting more points within each port vase. Sampling at different depths would complete the port landscape and likely provide a representative view of the present biota [61, 64].

Another important issue is the marker choice. In this particular study, the *COI* gene provided more OTU counts than 18S rDNA. however, most taxa detected from COI were planktonic microalgae and protozoans, while the 18S rDNA revealed the three invasive species. This result, however, cannot be extrapolated. Different studies have shown a greater utility of some Barcodes depending on the particular case study [10, 29, 65, 66, 67]. The complexity of marine communities would make it necessary to use two genes for a more complete view of diversity. Multiplexing allows for sequencing two genes simultaneously in the same run [68] and could be conduct in routine surveys at a cheaper cost and for faster results.

## Conclusion

An extremely simplified eDNA sampling methodology based on only three 1-L bottles of water per port, followed by NGS metabarcoding using *18S* rDNA and *COI* as genetic barcodes, in eight Bay of Biscay ports could detect three invasive invertebrates: the barnacle *Austrominius modestus*, the tubeworm *Ficopomatus enigmaticus* and the polychaete *Polydora triglanda*. The latter species occurred at very low density in visual inventory, despite minimal sampling efforts. The same species had been previously found in visual and DNA barcoding surveys in the same ports. Comparisons among the current costs of visual surveys, conventional barcoding and this simplified metabarcoding protocol indicate the use of metabarcoding for early biosecurity alerts would be beneficial.

## Supporting information

S1 Table Taxa found in each port with *18S* rDNA and *Cytochrome Oxidase I* metabarcodes. In red exotics species.
(DOCX)

S2 Table Taxa found from metabarcoding and barcoding in all the ports of the studied region. In red exotics species.
(DOCX)

S1 Fig Alpha rarefaction graphs found for *Cytochrome oxidase I* (a) and *18S* rDNA genes (b) using as metric Observed- species (OTUS) in water samples collected within Asturian ports (x-axis: read number; y-axis: number of OTUS). (DOCX)
