# Supplementary material for: DNA in a bottle—Rapid metabarcoding survey for early alerts of invasive species in ports
Source: PLoS One. 2017 Sep 5;12(9):e0183347. doi: 10.1371/journal.pone.0183347 (PMC5584753; doi:10.1371/journal.pone.0183347)
Supplement: S1 Table — In red exotics species. (DOCX) [file pone.0183347.s001.docx]

**S1 Table. Taxa found in each port with 18S rDNA and Cytochrome Oxidase I metabarcodes.** In red exotics species.

| Phylum | Class | Order | Family | Genus | Species | COI-Eo | 18S-Eo | COI-Luarca | 18S-Luarca | COI-Cudillero16 | COI-Aviles | 18S-Aviles | COI-Gijon | 18S-Gijon | COI-Villaviciosa16 | COI-Ribadesella | 18S-Ribadesella | COI-Llanes | 18S-Llanes |
| --- | --- | --- | --- | --- | --- | --- | --- | --- | --- | --- | --- | --- | --- | --- | --- | --- | --- | --- | --- |
| Oomycota | Bangiophyceae | Bangiales | Bangiaceae |  |  | 2 | 0 | 7 | 0 | 0 | 0 | 0 | 0 | 0 | 0 | 3 | 0 | 1 | 0 |
| Chlorophyta | prasinophytes | Mamiellales |  |  |  | 27 | 0 | 187 | 0 | 0 | 0 | 0 | 6 | 0 | 0 | 23 | 0 | 2 | 0 |
| Mollusca | Gastropoda |  | Trochidae |  |  | 0 | 0 | 0 | 0 | 0 | 0 | 0 | 7 | 0 | 0 | 0 | 0 | 0 | 0 |
| Annelida |  |  |  |  | Abarenicola | 0 | 0 | 0 | 0 | 0 | 0 | 0 | 0 | 0 | 0 | 0 | 0 | 0 | 7 |
| SAR | Rhizaria | Radiolaria | Acantharia |  | Acanthostaurus | 0 | 0 | 0 | 0 | 0 | 0 | 0 | 0 | 0 | 0 | 0 | 0 | 0 | 6 |
|  | Basidiomycota | Agaricomycotina |  |  | Agaricomycetes | 0 | 0 | 0 | 0 | 0 | 0 | 1 | 0 | 0 | 0 | 0 | 0 | 0 | 3 |
|  | Phaeophyceae | Laminariales | Alariaceae | Alaria | Alaria | 0 | 0 | 0 | 0 | 0 | 0 | 0 | 20 | 0 | 0 | 0 | 0 | 0 | 0 |
| Chordata | Actinopterygii | Albuliformes | Albulidae | Albula | Albula glossodonta | 89 | 0 | 0 | 0 | 0 | 244 | 0 | 6 | 0 | 41 | 0 | 0 | 0 | 0 |
|  | Alveolata | Protalveolata | Syndiniales |  | Amoebophrya sp. | 0 | 0 | 0 | 0 | 0 | 0 | 0 | 0 | 0 | 0 | 0 | 0 | 0 | 61 |
| Chordata | Craniata | Squamata | Amphisbaenidae | Amphisbaena | Amphisbaena | 14 | 0 | 1 | 0 | 0 | 0 | 0 | 0 | 0 | 0 | 0 | 0 | 0 | 0 |
|  | Ancyromonadida | Ancyromonas |  |  | Ancyromonas_micra | 0 | 0 | 0 | 0 | 0 | 0 | 0 | 0 | 0 | 0 | 0 | 0 | 0 | 5 |
| Nematoda | Chromadorea | Aphelenchida |  |  | Aphelenchoides sp | 0 | 0 | 0 | 0 | 0 | 0 | 0 | 0 | 0 | 0 | 0 | 0 | 0 | 5 |
|  | Stramenopiles | Labyrinthulomycetes | Thraustochytriaceae |  | Aplanochytrium sp | 0 | 0 | 0 | 0 | 0 | 0 | 3 | 0 | 0 | 0 | 0 | 0 | 0 | 5 |
| Alveolata | Apicomplexa | Conoidasida | Gregarinasina |  | Archigregarinorida | 0 | 0 | 0 | 1 | 0 | 0 | 0 | 0 | 5 | 0 | 0 | 0 | 0 | 21 |
|  |  | Entoprocta | Barentsiidae |  | Barentsia sp | 0 | 0 | 0 | 0 | 0 | 0 | 5 | 0 | 0 | 0 | 0 | 0 | 0 | 0 |
| Archaeplastida | Chloroplastida | Chlorophyta | Mamiellophyceae | Bathycoccus | Bathycoccus prasinos | 49 | 0 | 4 | 0 | 3 | 0 | 0 | 199 | 1 | 107 | 275 | 2 | 15 | 133 |
| Ochrophyta | Phaeophyceae | Fucales | Sargassaceae | Bifurcaria | Bifurcaria bifurcata | 0 | 0 | 0 | 0 | 11 | 0 | 0 | 0 | 0 | 0 | 0 | 0 | 0 | 0 |
|  | Alveolata | Dinoflagellata |  |  | Blastodinium_contortum | 0 | 0 | 0 | 0 | 0 | 0 | 0 | 0 | 0 | 0 | 0 | 0 | 0 | 4 |
| Annelida |  |  |  |  | Boccardiella_ligerica | 0 | 0 | 0 | 0 | 0 | 0 | 0 | 0 | 0 | 0 | 0 | 0 | 0 | 19 |
| Ascomycota | Pezizomycotina | Dothideomycetes |  |  | Boeremia | 0 | 0 | 0 | 0 | 0 | 0 | 10 | 0 | 0 | 0 | 0 | 0 | 0 | 1 |
| Annelida |  |  |  |  | Capitella | 0 | 9 | 0 | 0 | 0 | 0 | 0 | 0 | 0 | 0 | 0 | 0 | 0 | 28 |
| Gastrotricha |  |  | Chaetonotidae |  | Chaetonotus_sp. | 0 | 0 | 0 | 0 | 0 | 0 | 0 | 0 | 0 | 0 | 0 | 0 | 0 | 13 |
| Archaeplastida | Chloroplastida | Chlorophyta | Trebouxiophyceae | Chlorella | Chlorella_sorokiniana | 69 | 0 | 113 | 0 | 33 | 1 | 6 | 77 | 0 | 26 | 93 | 0 | 4 | 0 |
| Archaeplastida | Chloroplastida | Chlorophyta | Chlorophyceae | Chlamydomonas | Chlorococcum sp | 0 | 0 | 0 | 0 | 0 | 0 | 3.978 | 0 | 0 | 0 | 0 | 0 | 0 | 0 |
| Alveolata | Ciliophora | Intramacronucleata | Spirotrichea |  | Choreotrichia | 0 | 0 | 0 | 0 | 0 | 0 | 18 | 0 | 1 | 0 | 0 | 0 | 0 | 231 |
| Haptophyta | Prymnesiophyceae | Prymnesiales |  |  | Chrysochromulina | 0 | 0 | 0 | 0 | 0 | 0 | 0 | 0 | 0 | 0 | 0 | 0 | 0 | 11 |
| Chordata | Actinopterygii | Cypriniformes | Cyprinidae | Clinostomus | Clinostomus funduloides | 938 | 0 | 125 | 0 | 0 | 22 | 0 | 154 | 0 | 114 | 35 | 0 | 7 | 0 |
| Chordata | Ascidiacea | Stolidobranchia | Styelidae | Cnemidocarpa | Cnemidocarpa | 0 | 0 | 0 | 0 | 0 | 0 | 0 | 811 | 0 | 0 | 4 | 0 | 0 | 0 |
| Stramenopiles | Diatomea | Bacillariophytina | Bacillariophyceae |  | Cymbella | 0 | 6 | 0 | 0 | 0 | 0 | 0 | 0 | 0 | 0 | 0 | 0 | 0 | 0 |
| Chordata | Actinopterygii | Cypriniformes | Cyprinidae | Cyprinella | Cyprinella spiloptera | 0 | 0 | 0 | 0 | 17 | 0 | 0 | 0 | 0 | 0 | 0 | 0 | 0 | 0 |
| Archaeplastida | Chloroplastida | Chlorophyta | Chlorophyceae | | Desmodesmus sp | 0 | 0 | 0 | 0 | 0 | 0 | 12 | 0 | 0 | 0 | 0 | 0 | 0 | 0 |
| Streptophyta | Bryopsida | Dicranales | Dicranaceae | Dicranum | Dicranum scoparium | 3 | 0 | 0 | 0 | 3 | 0 | 0 | 3 | 0 | 0 | 9 | 0 | 0 | 0 |
| Phaeophyceae |  | Dictyotales | Dictyotaceae | Dictyopteris | Dictyopteris | 0 | 0 | 0 | 0 | 0 | 0 | 0 | 5 | 0 | 0 | 0 | 0 | 0 | 0 |
| Chordata | Chondrichthyes | Rajiformes | Rajidae | Dipturus | Dipturus | 0 | 0 | 0 | 0 | 0 | 0 | 0 | 6 | 0 | 0 | 0 | 0 | 0 | 0 |
| Chlorophyta | prasinophytes |  |  | Dolichomastix | Dolichomastix | 14 | 0 | 9 | 0 | 0 | 0 | 0 | 11 | 0 | 0 | 18 | 0 | 1 | 0 |
| Arthropoda | Crustacea | Maxillopoda |  |  | Elminius | 0 | 0 | 0 | 0 | 0 | 0 | 20 | 0 | 0 | 0 | 0 | 0 | 0 | 0 |
| Haptophyta | Prymnesiophyceae | Isochrysidales |  |  | Emiliania_huxleyi | 0 | 0 | 0 | 0 | 0 | 0 | 1 | 0 | 0 | 0 | 0 | 0 | 0 | 32 |
| Alveolata | Apicomplexa | Conoidasida | Gregarinasina |  | Eugregarinorida | 0 | 0 | 0 | 0 | 0 | 0 | 0 | 0 | 7 | 0 | 0 | 0 | 0 | 0 |
| Alveolata | Ciliophora | Intramacronucleata | Spirotrichea |  | Euplotia | 0 | 0 | 0 | 0 | 0 | 0 | 0 | 0 | 0 | 0 | 0 | 0 | 0 | 13 |
| Arthropoda | Malacostraca | Amphipoda | Lysianassidae | Eurythenes | Eurythenes gryllus | 3 | 0 | 0 | 0 | 16 | 0 | 0 | 1 | 0 | 16 | 15 | 0 | 0 | 0 |
| Annelida | Polychaeta |  | Serpulidae |  | Ficopomatus_enigmaticus | 0 | 0 | 0 | 0 | 0 | 0 | 0 | 0 | 0 | 0 | 0 | 0 | 0 | 6 |
| Bacillariophyta | Bacillariophyceae | Naviculales | Amphipleuraceae | Frustulia | Frustulia crassinervia/saxonica | 534 | 0 | 848 | 0 | 913 | 6 | 0 | 2962 | 0 | 689 | 454 | 0 | 273 | 0 |
|  | Cryptophyceae | Cryptomonadales |  |  | Geminigera_cryophila | 0 | 0 | 0 | 0 | 0 | 0 | 0 | 0 | 0 | 0 | 0 | 0 | 0 | 4 |
|  | Raphidophyceae | Chattonellales | Vacuolariaceae | Gonyostomum | Gonyostomum semen | 1 | 0 | 0 | 0 | 1 | 2 | 0 | 1 | 0 | 5 | 4 | 0 | 0 | 0 |
| Bacillariophyta | Fragilariophyceae | Fragilariales | Fragilariaceae | Grammonema | Grammonema | 2 | 0 | 1 | 0 | 0 | 1 | 0 | 1 | 0 | 0 | 1 | 0 | 0 | 0 |
| Alveolata | Apicomplexa | Conoidasida |  |  | Gregarinasina | 0 | 0 | 0 | 0 | 0 | 0 | 0 | 0 | 0 | 0 | 0 | 0 | 0 | 12 |
| Alveolata | Dinoflagellata | Dinophyceae |  |  | Gymnodinium | 0 | 0 | 0 | 0 | 0 | 0 | 0 | 0 | 0 | 0 | 0 | 1 | 0 | 29 |
| Alveolata | Dinoflagellata | Dinophyceae |  |  | Gyrodinium | 0 | 1 | 0 | 2 | 0 | 0 | 88 | 0 | 1 | 0 | 0 | 0 | 0 | 134 |
| Alveolata | Ciliophora | Intramacronucleata |  |  | Haptoria | 0 | 0 | 0 | 0 | 0 | 0 | 7 | 0 | 0 | 0 | 0 | 0 | 0 | 8 |
|  | Microsporidia |  |  |  | Hazardia_milleri | 0 | 0 | 0 | 0 | 0 | 0 | 0 | 0 | 7 | 0 | 0 | 0 | 0 | 0 |
| Echinodermata | Echinoidea | Echinoida | Echinometridae | Heliocidaris | Heliocidaris crassispina | 4 | 0 | 0 | 0 | 0 | 0 | 0 | 0 | 0 | 7 | 1008 | 0 | 0 | 0 |
| Alveolata | Ciliophora | Intramacronucleata | Spirotrichea |  | Hypotrichia | 0 | 0 | 0 | 0 | 0 | 0 | 0 | 0 | 0 | 0 | 0 | 0 | 0 | 9 |
| Chlorophyta | Chlorophyceae | Sphaeropleales | Selenastraceae | Kirchneriella | Kirchneriella aperta | 31 | 0 | 0 | 0 | 18 | 0 | 0 | 39 | 0 | 5 | 0 | 0 | 0 | 0 |
| Cnidaria | Anthozoa | Alcyonacea | Alcyoniidae | Klyxum | Klyxum | 17 | 0 | 0 | 0 | 0 | 0 | 0 | 0 | 0 | 8 | 0 | 0 | 0 | 0 |
| Mollusca | Gastropoda | __ | Lacunidae | Lacuna | Lacuna sp. | 3 | 0 | 0 | 0 | 1 | 0 | 0 | 7 | 0 | 0 | 11 | 0 | 0 | 0 |
| Phaeophyceae |  | Fucales | Sargassaceae | Landsburgia | Landsburgia | 0 | 0 | 0 | 0 | 0 | 0 | 0 | 5 | 0 | 0 | 0 | 0 | 0 | 0 |
| Alveolata | Ciliophora | Intramacronucleata | Litostomatea |  | Mesodiniidae | 0 | 6 | 0 | 0 | 0 | 0 | 0 | 0 | 0 | 0 | 0 | 0 | 0 | 10 |
| Chlorophyta | prasinophytes | Mamiellales | __ | Micromonas | Micromonas | 65 | 1 | 0 | 1 | 16 | 1 | 0 | 258 | 1 | 93 | 286 | 1 | 8 | 436 |
| Mollusca | Bivalvia |  |  |  | Mytiloidea | 0 | 0 | 0 | 0 | 0 | 0 | 0 | 0 | 0 | 0 | 0 | 0 | 0 | 15 |
| Ochrophyta | Phaeophyceae | |  |  | Nannochloris sp | 0 | 0 | 0 | 0 | 0 | 0 | 0 | 0 | 0 | 0 | 0 | 0 | 0 | 19 |
|  | Eustigmatophyceae | Eustigmatales | Monodopsidaceae | Nannochloropsis | Nannochloropsis salina | 89 | 0 | 141 | 0 | 72 | 15 | 0 | 37 | 0 | 9 | 37 | 0 | 1 | 0 |
| Ochrophyta | Bacillariophyceae | Naviculales | Naviculaceae | Navicula | Navicula | 0 | 0 | 0 | 0 | 0 | 0 | 0 | 0 | 0 | 0 | 0 | 0 | 0 | 15 |
| Oomycota |  | Arcellinida | Hyalospheniidae | Nebela | Nebela | 0 | 0 | 4 | 0 | 0 | 0 | 0 | 5 | 0 | 0 | 3 | 0 | 0 | 0 |
| Bacillariophyta | Bacillariophyta | Bacillariophyceae | Bacillariaceae | Nitzschia | Nitzschia palea | 0 | 0 | 0 | 0 | 0 | 0 | 0 | 166 | 0 | 0 | 0 | 0 | 0 | 0 |
|  |  |  |  |  | Odontella | 0 | 0 | 0 | 0 | 0 | 0 | 0 | 0 | 0 | 0 | 0 | 0 | 0 | 62 |
|  |  |  |  |  | Oligohymenophorea | 0 | 0 | 0 | 0 | 0 | 0 | 0 | 0 | 0 | 0 | 0 | 0 | 0 | 368 |
|  |  |  |  |  | Oligotrichia | 0 | 0 | 0 | 0 | 0 | 0 | 118 | 0 | 0 | 0 | 0 | 0 | 0 | 1.396 |
| Chordata | Actinopterygii | Cypriniformes | Cyprinidae | Oregonichthys | Oregonichthys kalawatseti | 1521 | 0 | 0 | 0 | 86 | 29 | 0 | 1450 | 0 | 1335 | 32 | 0 | 6 | 0 |
|  | Chloroplastida | Chlorophyta | Mamiellophyceae |  | Ostreococcus sp | 0 | 6 | 0 | 21 | 0 | 0 | 2 | 0 | 2 | 0 | 0 | 4 | 0 | 596 |
| Stramenopiles | Chrysophyceae | Ochromonadales | Paraphysomonas |  | Paraphysomonas sp | 0 | 0 | 0 | 0 | 0 | 0 | 0 | 0 | 0 | 0 | 0 | 0 | 0 | 25 |
| Oomycota | Peronospea | Peronosporales | Peronosporaceae | Peronospora | Peronospora cerastii | 0 | 0 | 0 | 0 | 2 | 1 | 0 | 4 | 0 | 0 | 2 | 0 | 0 | 0 |
| Alveolata | Ciliophora | Intramacronucleata | Conthreep |  | Phyllopharyngea | 0 | 0 | 0 | 4 | 0 | 0 | 0 | 0 | 0 | 0 | 0 | 0 | 0 | 28 |
| Oomycota | Peronospea | Peronosporales | __ | Phytophthora | Phytophthora bilorbang | 15 | 0 | 0 | 0 | 11 | 4 | 0 | 13 | 0 | 10 | 3 | 0 | 0 | 0 |
| Bacillariophyta | Bacillariophyceae | Naviculales | Pinnulariaceae | Pinnularia | Pinnularia | 1 | 0 | 5 | 0 | 0 | 0 | 0 | 0 | 0 | 0 | 17 | 0 | 1 | 0 |
| Echinodermata | Asteroidea | Forcipulatida | Asteriidae | Pisaster | Pisaster ochraceus | 2 | 0 | 0 | 0 | 23 | 0 | 0 | 0 | 0 | 2 | 4 | 0 | 6 | 0 |
| Annelida |  |  |  |  | Polydora | 0 | 0 | 0 | 0 | 0 | 0 | 0 | 0 | 0 | 0 | 0 | 0 | 0 | 8 |
| Alveolata | Ciliophora | Intramacronucleata | Conthreep |  | Prostomatea | 0 | 0 | 0 | 0 | 0 | 0 | 10 | 0 | 0 | 0 | 0 | 0 | 0 | 10 |
| Rhizaria | Cercozoa | Thecofilosea | Cryomonadida |  | Protaspidae | 0 | 0 | 0 | 0 | 0 | 0 | 0 | 0 | 0 | 0 | 0 | 0 | 0 | 4 |
| Stramenopiles | Diatomea | Bacillariophytina | Bacillariophyceae |  | Psammodictyon | 0 | 0 | 0 | 0 | 0 | 0 | 0 | 0 | 0 | 0 | 0 | 0 | 0 | 4 |
| Oomycota | Peronospea | Peronosporales | Peronosporaceae | Pseudoperonospora | Pseudoperonospora humuli | 1 | 0 | 0 | 0 | 2 | 0 | 0 | 3 | 0 | 0 | 3 | 0 | 0 | 0 |
|  | Chloroplastida | Chlorophyta | Prasinophytae | Pyramimonas | Pyramimonas sp | 0 | 0 | 0 | 0 | 0 | 0 | 12 | 0 | 0 | 0 | 0 | 0 | 0 | 17 |
| Oomycota | Peronospea | Pythiales | Pythiaceae | Pythium | Pythium | 13 | 0 | 16 | 2 | 24 | 1 | 0 | 16 | 0 | 5 | 15 | 0 | 1 | 0 |
| Cryptophyceae | Cryptomonadales |  |  |  | Rhodomonas sp | 0 | 0 | 0 | 0 | 0 | 0 | 0 | 0 | 0 | 0 | 0 | 0 | 0 | 10 |
| Phaeophyceae | __ | Ectocarpales | Scytosiphonaceae | Scytosiphon | Scytosiphon lomentaria | 35 | 0 | 1 | 0 | 0 | 1 | 0 | 1102 | 0 | 0 | 1 | 0 | 0 | 0 |
| Mollusca | Cephalopoda | Sepiolida | Sepiolidae | Sepietta | Sepietta | 332 | 0 | 6 | 0 | 404 | 141 | 0 | 508 | 0 | 136 | 97 | 0 | 23 | 0 |
| Annelida | Polychaeta | Sabellida | Serpulidae | Serpula | Serpula | 0 | 0 | 0 | 0 | 0 | 0 | 0 | 8 | 0 | 0 | 0 | 0 | 0 | 0 |
| Stramenopiles | Diatomea | Bacillariophytina | Mediophyceae |  | Skeletonema | 0 | 0 | 0 | 0 | 0 | 0 | 37 | 0 | 0 | 0 | 0 | 0 | 0 | 28 |
| Arthropoda | Malacostraca | Decapoda | Solenoceridae | Solenocera | Solenocera | 1 | 0 | 6 | 0 | 0 | 0 | 0 | 4 | 0 | 0 | 2 | 0 | 0 | 0 |
| Ascomycota | Pezizomycotina |  |  |  | Sordariomycetes | 0 | 0 | 0 | 0 | 0 | 0 | 25 | 0 | 0 | 0 | 0 | 0 | 0 | 0 |
| Phaeophyceae | __ | Scytothamnales | __ | Stereocladon | Stereocladon rugulosus | 1644 | 0 | 585 | 0 | 9 | 141 | 0 | 350 | 0 | 1 | 102 | 0 | 37 | 0 |
| Mollusca | Gastropoda | __ | Strombidae | Strombus | Strombus aurisdianae | 4 | 0 | 0 | 0 | 5 | 0 | 0 | 12 | 0 | 1 | 0 | 0 | 0 | 0 |
| Cryptophyceae | Cryptomonadales |  |  |  | Teleaulax sp | 0 | 0 | 0 | 0 | 0 | 0 | 12 | 0 | 0 | 0 | 0 | 0 | 0 | 23 |
| Gastrotricha | __ | Macrodasyida | Thaumastodermatidae | Tetranchyroderma | Tetranchyroderma quadritentaculatum | 164 | 0 | 324 | 0 | 0 | 59 | 0 | 389 | 0 | 0 | 62 | 0 | 4 | 0 |
| Chloroplastida | Chlorophyta | Chlorodendrales |  |  | Tetraselmis sp | 0 | 0 | 0 | 0 | 0 | 0 | 0 | 0 | 0 | 0 | 0 | 0 | 0 | 13 |
| Bacillariophyta | Coscinodiscophyceae |  | Thalassiosiraceae | Thalassiosira | Thalassiosira | 0 | 16 | 0 | 0 | 0 | 0 | 94 | 16 | 0 | 0 | 0 | 3 | 0 | 24 |
| Chloroplastida | Chlorophyta | Ulvophyceae |  |  | Ulva sp | 0 | 0 | 0 | 0 | 0 | 0 | 0 | 0 | 0 | 0 | 0 | 0 | 0 | 4 |
| Oomycota | Peronospea | Mucorales | __ | Umbelopsis | Umbelopsis ramanniana | 0 | 0 | 0 | 0 | 9 | 0 | 0 | 0 | 0 | 0 | 0 | 0 | 0 | 0 |
| Annelida |  |  |  |  | Vermiliopsis | 0 | 0 | 0 | 0 | 0 | 0 | 0 | 0 | 0 | 0 | 0 | 0 | 0 | 4 |
| Arthropoda | Maxillopoda | Sessilia | Balanidae | Wanella | Wanella | 3 | 0 | 2 | 0 | 0 | 0 | 0 | 0 | 0 | 0 | 0 | 0 | 0 | 0 |
| Arthropoda | Malacostraca | Isopoda | Desmosomatidae | |  | 50 | 0 | 0 | 0 | 19 | 23 | 0 | 64 | 0 | 5 | 9 | 0 | 0 | 0 |
| Phaeophyceae | __ | Ectocarpales | Acinetosporaceae | |  | 34 | 0 | 0 | 0 | 9 | 0 | 0 | 1 | 0 | 1 | 0 | 0 | 0 | 0 |
| Phaeophyceae | __ | Ectocarpales | Chordariaceae | |  | 7 | 0 | 0 | 0 | 0 | 17 | 0 | 0 | 0 | 1 | 1 | 0 | 0 | 0 |
|  |  |  |  |  | **TOTAL** | 5781 | 45 | 2385 | 31 | **1707** | **709** | 4459 | 8727 | 25 | **2617** | **2629** | **11** | **390** | 3919 |
