# Supplementary material for: DNA in a bottle—Rapid metabarcoding survey for early alerts of invasive species in ports
Source: PLoS One. 2017 Sep 5;12(9):e0183347. doi: 10.1371/journal.pone.0183347 (PMC5584753; doi:10.1371/journal.pone.0183347)
Supplement: S2 Table — In red exotics species. (DOCX) [file pone.0183347.s002.docx]

**S2 Table.** **Taxa found from metabarcoding and barcoding in all the ports of the studied region**. In red exotics species.

| Taxonomy | **Species** | **Fouling** | Metabarcoding OTUs | Proportion of barcodes (n=671) |
| --- | --- | --- | --- | --- |
| Annelida | *Abarenicola spp.* | Yes | 1 |  |
| Mollusca, Neoloricata, Acanthochitonidae | *Acanthochitona crinita* | *Yes* | 0 | 0.00105042 |
| Chordata, Actinopterygii, Albulidae | *Albula glossodonta* | No | 1 | 0 |
| Bryozoa, Gymnolaemata, Vesiculariidae | *Amathia imbricata* | *Yes* | 0 | 0.00105042 |
| Bryozoa, Gymnolaemata, Vesiculariidae | *Amathia verticillata* | *Yes* | 0 | 0.007352941 |
| Arthropoda, Maxillopoda, Balanidae | *Amphibalanus amphitrite* | *Yes* | 0 | 0.005252101 |
| Arthropoda, Maxillopoda, Balanidae | *Amphibalanus eburneus* | *Yes* | 0 | 0.004201681 |
| Chordata, Squamata, Amphisbaenidae | *Amphisbaena* | No | 1 | 0 |
| Arthropoda, Malacostraca, Ampithoidae | *Ampithoe rubricata* | *Yes* | 0 | 0.00105042 |
| Cnidaria, Anthozoa, Actiniidae | *Anemonia sp.* | *Yes* | 0 | 0.00105042 |
| Nematoda, Chromadorea | *Aphelenchoides sp* | Yes | 1 | 0 |
| Chordata, Ascidiacea, Ascidiidae | *Ascidiella aspersa* | *Yes* | 0 | 0.003151261 |
| Entoprocta, Barentsiidae | *Barentsia sp* | Yes | 1 | 0 |
| Annelida | *Boccardiella_ligerica* | Yes | 1 | 0 |
| Chordata, Ascidiacea, Styelidae | *Botrylloides violaceus* | *Yes* | 0 | 0.004201681 |
| Chordata, Ascidiacea, Styelidae | *Botryllus schlosseri* | *Yes* | 0 | 0.00210084 |
| Bryozoa, Gymnolaemata, Bugulidae | *Bugula neritina* | *Yes* | 0 | 0.006302521 |
| Porifera, Demospongiae, Callyspongiidae | *Callyspongia siphonella* | *Yes* | 0 | 0.00105042 |
| Annelida | *Capitella spp.* | Yes | 1 | 0 |
| Gastrotricha, Chaetonotidae | *Chaetonotus_sp.* | Yes | 1 | 0 |
| Chordata, Aves, Anatidae | *Chloephaga picta* | *No* | 1 | 0 |
| Arthropoda, Cirripedia, Chthamalidae | *Chthamalus montagui* | *Yes* | 0 | 0.015756303 |
| Arthropoda, Cirripedia, Chthamalidae | *Chthamalus stellatus* | *Yes* | 0 | 0.006302521 |
| Mollusca, Gastropoda, Rissoidae | *Cingula trifasciata* | *Yes* | 0 | 0.00105042 |
| Arthropoda, Malacostraca, Diogenidae | *Clibanarius erythropus* | *Yes* | 0 | 0.00105042 |
| Chordata, Actinopterygii, Cyprinidae | *Clinostomus funduloides* | *No* | 1 | 0 |
| Chordata, Ascidiacea, Styelidae | *Cnemidocarpa* | *Yes* | 1 | 0 |
| Mollusca, Bivalvia, Ostreidae | *Crassostrea gigas* | *Yes* | 0 | 0.047268908 |
| Mollusca, Gastropoda, Calyptraeidae | *Crepidula fornicata* | *Yes* | 0 | 0.00105042 |
| Bryozoa, Gymnolaemata, Cryptosulidae | *Cryptosula pallasiana* | *Yes* | 0 | 0.00105042 |
| Chordata, Actinopterygii, Cyprinidae | *Cyprinella spiloptera* | *No* | 1 | 0 |
| Chordata, Chondrichthyes, Rajidae | *Dipturus spp.* | *No* | 1 | 0 |
| **Arthropoda, Cirripedia, Chthamalidae** | ***Austrominius (Elminius) modestus*** | **Yes** | **1** | 0.015756303 |
| Nemertea, Enopla, Emplectonematidae | *Emplectonema gracile* | *Yes* | 0 | 0.003151261 |
| Arthropoda, Malacostraca, Lyssianasidae | *Eurythenes gryllus* | Yes | 1 | 0 |
| Arthropoda, Malacostraca, Hyalidae | *Fam. Hyalidae* | *Yes* | 0 | 0.007352941 |
| Mollusca, Gastropoda, Chromodorididae | *Felimare villafranca* | *Yes* | 0 | 0.00210084 |
| **Annelida, Polychaeta, Serpulidae** | ***Ficopomatus enigmaticus*** | ***Yes*** | **1** | 0.056722689 |
| Mollusca, Gastropoda, Trochidae | *Gibbula umbilicalis* | *Yes* | 0 | 0.046218487 |
| Annelida, Polychaeta, Nereididae | *Hediste diversicolor* | *Yes* | 0 | 0.00105042 |
| Echinodermata, Echinoidea, Echinometridae | *Heliocidaris crassispina* | Yes | 1 | 0 |
| Porifera, Demospongia, Halichondriidae | *Hymeniacidon perlevis* | *Yes* | 0 | 0.00210084 |
| Porifera, Demospongia, Halichondriidae | *Hymeniacidon sp.* | *Yes* | 0 | 0.004201681 |
| Mollusca, Gastropoda, Discodorididae | *Jorunna tomentosa* | *Yes* | 0 | 0.00105042 |
| Cnidaria, Anthozoa, Alcyoniidae | *Klyxum* | Yes | 1 | 0 |
| Mollusca, Gastropoda, Lacunidae | *Lacuna sp.* | Yes | 1 | 0 |
| Annelida, Polychaeta, Spionidae | *Laonice cirrata* | *Yes* | 0 | 0.00105042 |
| Mollusca, Bivalvia, Lasaeidae | *Lasaea adansoni* | *Yes* | 0 | 0.00210084 |
| Annelida, Polychaeta, Eunicidae | *Leodice harassii* | *Yes* | 0 | 0.00105042 |
| Mollusca, Polyplacophora, Lepidochitonidae | *Lepidochitona cinerea* | *Yes* | 0 | 0.00105042 |
| Mollusca, Gastropoda, Littorinidae | *Littorina saxatilis* | *Yes* | 0 | 0.004201681 |
| Mollusca, Gastropoda, Littorinidae | *Littorina sp.* | *Yes* | 0 | 0.00105042 |
| Arthropoda, Malacostraca, Cymothoidae | *Livoneca redmanii* | *Yes* | 0 | 0.005252101 |
| Mollusca, Gastropoda, Littorinidae | *Melarhaphe neritoides* | *Yes* | 0 | 0.00210084 |
| Chordata, Ascidiacea, Pyuridae | *Microcosmus squamiger* | *Yes* | 0 | 0.004201681 |
| Arthropoda, Malacostraca, Corophiidae | *Monocorophium insidiosum* | *Yes* | 0 | 0.006302521 |
| Chordata, Ascidiacea, Polyclinidae | *Morchellium argus* | *Yes* | 0 | 0.00105042 |
| Mollusca, Bivalvia, Mytilidae | *Mytilaster minimus* | *Yes* | 0 | 0.022058824 |
| Mollusca, Bivalvia, Mytilidae | Mytilidae | Yes | 1 | 0 |
| Mollusca, Bivalvia, Mytilidae | *Mytilus edulis* | *Yes* | 0 | 0.003151261 |
| Mollusca, Bivalvia, Mytilidae | *Mytilus galloprovincialis* | *Yes* | 0 | 0.048319328 |
| Mollusca, Bivalvia, Mytilidae | *Mytilus sp.* | *Yes* | 0 | 0.134453782 |
| Mollusca, Bivalvia, Mytilidae | *Mytilus trossulus* | *Yes* | 0 | 0.013655462 |
| Mollusca, Bivalvia, Mytillidae | *Xenostrobus securis* | *Yes* | 0 | 0.055672269 |
| Mollusca, Gastropoda, Nassariidae | *Nassarius incrassatus* | *Yes* | 0 | 0.00210084 |
| Annelida, Polychaeta, Nereididae | *Neanthes fucata* | *Yes* | 0 | 0.00210084 |
| Annelida, Polychaeta, Nereididae | *Nereis falsa* | *Yes* | 0 | 0.00210084 |
| Cnidaria, Hydrozoa, Campanulariidae | *Obelia geniculata* | *Yes* | 0 | 0.00105042 |
| Echinodermata, Ophiouroidea, Ophiotrichidae | *Ophiothrix sp.* | *Yes* | 0 | 0.00105042 |
| Chordata, Actinopterygii, Cyprinidae | *Oregonichthys kalawatseti* | *No* | 1 | 0 |
| Mollusca, Bivalvia, Ostreidae | *Ostrea edulis* | *Yes* | 0 | 0.003151261 |
| Mollusca, Bivalvia, Ostreidae | *Ostrea stentina* | *Yes* | 0 | 0.00105042 |
| Arthropoda, Malacostraca, Palaemonidae | *Palaemon elegans* | *Yes* | 0 | 0.003151261 |
| Arthropoda, Malacostraca, Palaemonidae | *Palaemon serratus* | *Yes* | 0 | 0.00105042 |
| Echinodermata, Echinoidea, Parechinidae | *Paracentrotus lividus* | *Yes* | 0 | 0.00105042 |
| Mollusca, Gastropoda, Patellidae | *Patella aspera* | *Yes* | 0 | 0.005252101 |
| Mollusca, Gastropoda, Patellidae | *Patella depressa* | *Yes* | 0 | 0.011554622 |
| Mollusca, Gastropoda, Patellidae | *Patella vulgata* | *Yes* | 0 | 0.033613445 |
| Arthropoda, Cirripedia, Balanidae | *Perforatus perforatus* | *Yes* | 0 | 0.003151261 |
| Mollusca, Gastropoda, Hydrobiidae | *Peringia ulvae* | *Yes* | 0 | 0.00105042 |
| Mollusca, Gastropoda, Trochidae | *Phorcus articulatus* | *Yes* | 0 | 0.00210084 |
| Mollusca, Gastropoda, Trochidae | *Phorcus lineatus* | *Yes* | 0 | 0.016806723 |
| Arthropoda, Malacostraca, Pilumnidae | *Pilumnus hirtellus* | *Yes* | 0 | 0.00105042 |
| Echinodermata, Asteroidea, Asteriidae | *Pisaster ochraceus* | Yes | 1 | 0 |
| Annelida, Polychaeta, Nereididae | *Platynereis dumerilii* | *Yes* | 0 | 0.025210084 |
| **Annelida, Polychaeta, Spionidae** | ***Polydora triglanda*** | **Yes** | **1** | 0.00105042 |
| Echinodermata, Echinoidea, Parechinidae | *Psammechinus miliaris* | *Yes* | 0 | 0.00210084 |
| Nemertea, Anopla, Lineidae | *Riseriellus occultus* | *Yes* | 0 | 0.00210084 |
| Mollusca, Cephalopoda, Sepiolidae | *Sepietta sp.* | *No* | 1 | 0 |
| Annelida, Polychaeta, Serpulidae | *Serpula sp.* | *Yes* | 1 | 0 |
| Arthropoda, Malacostraca, Solenoceridae | *Solenocera sp.* | Yes | 1 | 0 |
| Arthropoda, Malacostraca, Sphaeromatidae | *Sphaeroma serratum* | *Yes* | 0 | 0.00105042 |
| Annelida, Polychaeta, Serpulidae | *Spirobranchus triqueter* | *Yes* | 0 | 0.00210084 |
| Mollusca, Gastropoda, Strombidae | *Strombus aurisdianae* | *Yes* | 1 | 0 |
| Chordata, Ascidiacea, Styelidae | *Styela clava* | *Yes* | 0 | 0.00105042 |
| Chordata, Ascidiacea, Styelidae | *Styela plicata* | *Yes* | 0 | 0.003151261 |
| Annelida, Polychaeta, Syllidae | *Syllis gracilis* | *Yes* | 0 | 0.00105042 |
| Annelida, Polychaeta, Syllidae | *Syllis sp.* | *Yes* | 0 | 0.00210084 |
| Annelida, Polychaeta, Terebellidae | *Terebella lapidaria* | *Yes* | 0 | 0.00105042 |
| Gastrotricha, Thaumastodermatidae | *Tetranchyroderma quadritentaculatum* | Yes | 1 | 0 |
| Annelida, Polychaeta, Serpulidae | *Vermiliopsis striaticeps* | Yes | 1 | 0.00105042 |
| Arthropoda, Maxillopoda, Balanidae | *Wanella spp.* | *Yes* | 1 | 0 |
| **Bryozoa, Gymnolaemata, Watersiporidae** | ***Watersipora subtorquata*** | ***Yes*** | **0** | **0.011554622** |
