# Supplementary material for: DNA in a bottle—Rapid metabarcoding survey for early alerts of invasive species in ports
Source: PLoS One. 2017 Sep 5;12(9):e0183347. doi: 10.1371/journal.pone.0183347 (PMC5584753; doi:10.1371/journal.pone.0183347)
Supplement: S1 Fig — (DOCX) [file pone.0183347.s003.docx]

**S1 Figure**. **Alpha rarefaction graphs found for *Cytochrome oxidase I* (a) and *18S* rDNA genes (b) using as metric Observed- species (OTUS) in water samples collected within Asturian ports** (x-axis: read number; y-axis: number of OTUS).


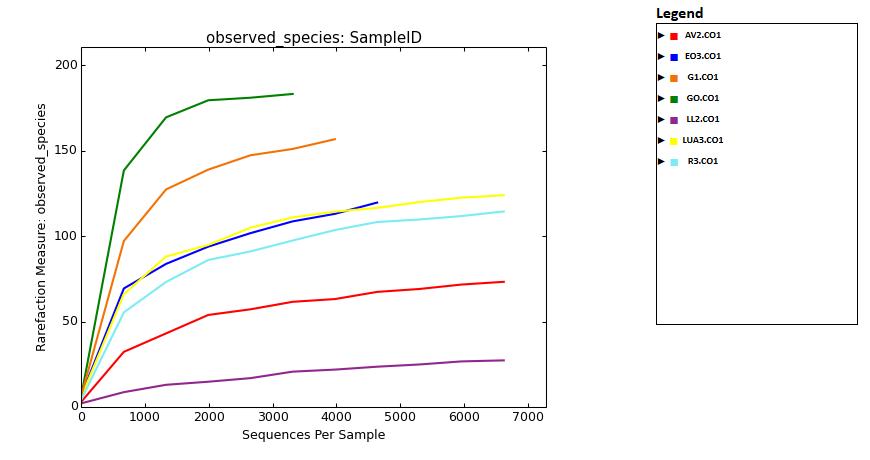

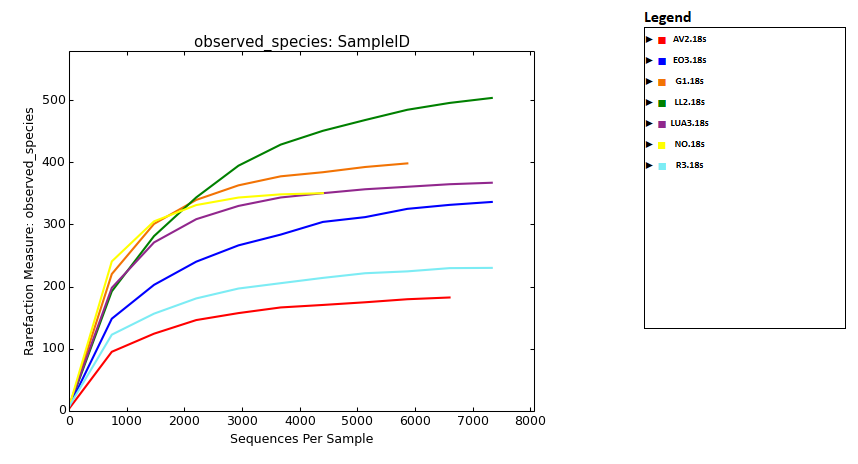


a)

b)
